# Preventing Child Sexual Abuse and the Use of Child Sexual Abuse Materials: Following up on the German Prevention Project *Dunkelfeld*

**DOI:** 10.1007/s10935-024-00792-0

**Published:** 2024-09-13

**Authors:** Klaus M. Beier, Julia Nentzl, Maximilian von Heyden, Mariam Fishere, Till Amelung

**Affiliations:** Charité – Universitätsmedizin Berlin, corporate member of Freie Universität Berlin and Humboldt-Universität zu Berlin, Institut für Sexualwissenschaft und Sexualmedizin, Luisenstraße 57, 10117 Berlin, Germany

**Keywords:** Pedophilic disorder, Hebephilic disorder, Child sexual exploitation material, Kein Täter werden, Public health approach

## Abstract

Sexual interest in minors (i.e., pedophilia and hebephilia) is considered a risk factor for Child Sexual Abuse (CSA) and the use of Child Sexual Abuse Material (CSAM). This study examined the long-term development of CSA, CSAM use, associated cognitions, and quality of life among self-referred, help-seeking individuals diagnosed with pedophilic or hebephilic disorder (PHD) outside the judicial system. Of the 110 eligible men who had undergone therapy for PHD due to psychological distress or risk of offending, 56 were available for follow-up assessment 1–11 years after treatment. Behavioral manifestations, offense-supportive cognitions, and quality of life were evaluated using interviews and psychometric measures. At follow-up, 7.7% of participants with a history of CSA had re-offended, whereas 89.1% of previous CSAM users reported recidivism, although with less severe material. No new CSA offenses occurred among those without prior CSA. Treatment led to increased cognitive victim empathy and reductions in CSA-supportive and CSAM-supportive attitudes, but only the latter improvement persisted through follow-up. Participants exhibited elevated CSA-supportive attitudes relative to community norms at all time points and diminished quality of life at follow-up. Pedophilic and hebephilic disorder carry a persistent risk of sexual offending, particularly regarding CSAM use. Therapeutic gains in offense-supportive cognitions may erode over time without ongoing care. A comprehensive public health approach encompassing early detection, prevention, and expanded treatment access that addresses both the risk of reoffending and overall well-being is crucial for reducing sexual victimization and improving mental health outcomes for individuals from this target group.

## Introduction

Globally, approximately 18% of girls and 8% of boys under 18 experience unwanted sexual contact with an adult (child sexual abuse, CSA), with studies relying on self-reports revealing prevalence rates more than 30 times greater than studies relying on official reports by child protection services or police (Stoltenborgh et al., 2011), leaving many offenders undetected. Recognized as a public health crisis (Cant et al., 2022), CSA has significant long-term effects (Hailes et al., 2019).

The Internet’s growth exacerbates CSA cases by facilitating the spread of media depicting child sexual abuse (child sexual abuse material, CSAM) (Seigfried-Spellar & Soldino, 2019). Victims of documented CSA suffer from enduring trauma as the documentation of the sexual abuse inflicted upon them is made public and continues to be accessible (Weiler et al., 2010). In 2021, 72% of CSAM detected by the Internet Watch Foundation were traced to Europe, of which 3% were hosted in Germany. Additionally, a 7% increase in CSAM use was recorded in Germany, with 42,075 registered cases of dissemination, acquisition, and possession, more than double the number of CSA offenses recorded that year (Bundeskriminalamt, 2022). Yet, much like CSA offenders, CSAM users continue to escape detection (Insoll et al., 2022).

Sexual interest in prepubescent and early pubescent children is considered a risk factor for CSA and CSAM offenses (Seto et al., 2023), especially if the interest is exclusive (Beier, 1998; Eher et al., 2015). Sexual interest in prepubescent children, known as *pedophilia*, and interest in early pubescent children, referred to as *hebephilia*, are surprisingly prevalent in the general community. Prevalence estimates for self-reported sexual interest in children vary across studies, with some suggesting that the prevalence in the general adult male population is in a range of 1.7–5% (Bártová et al., 2021; Dombert et al., 2016). A review of 30 studies revealed a prevalence rate of sexual interest in children ranging from 2 to 24%, although most studies had poor external validity and a high risk of bias (Savoie et al., 2021). This paper uses the term PH to refer to both pedophilia and hebephilia, and PHD to denote the corresponding pedophilic or hebephilic disorders. Epidemiological studies have shown that only some individuals with PH sexual interests act upon their sexual impulses (see Savoie et al., 2021). For a diagnosis of a pedophilic or hebephilic disorder (other specified paraphilic disorder), both the DSM-5 (American Psychiatric Association, APA, 2013) and ICD-11 (WHO, 2019) stipulate not only the presence of said sexual interest but also personal distress, interpersonal difficulties, and the manifestation of sexual offenses against children or a risk of committing them. In summary, attraction to children alone is neither a criminal offense nor a diagnosable disorder, and not every individual with PH necessarily sexually abuses children. The condition, however, places increased demands on self-regulation and may be associated with substantial distress even in the absence of the offending behavior (Cohen et al., 2020; Lievesley et al., 2020). Globally, a broadening of the response is taking place, from punishing offenders through criminal justice to implementing proactive strategies that prevent at-risk individuals from offending in the first place (Cant et al., 2022).

### Preventive Approaches to CSA and Use of CSAM

The increasing global spread of child sexual abuse (CSA) and the proliferation of child sexual abuse material (CSAM) online necessitates a public health approach to prevention that extends beyond the judicial system. Directive 2011/92/EU calls for a comprehensive strategy encompassing offender prosecution, victim protection, and prevention. However, prevention efforts outside the judicial context often lack uniform terminology and a coherent framework.

Several calls for a comprehensive public health approach to CSA prevention have been made in the past (Letourneau et al., 2014). A comprehensive public health approach to CSA and CSAM prevention should incorporate universal, selective, and indicated strategies that address risk factors and promote protective factors at various levels. Focusing on risk factors for perpetration, universal prevention could begin in the primary school and family setting and focus on interventions like social skills training, which have been shown to lower the risk of antisocial development (Beelmann & Lösel, 2021). Furthermore, improving literacy about healthy sexual behavior and norms in secondary school may reduce the risk of harmful sexual conduct (Letourneau et al., 2022). Selective prevention could address high-risk groups with targeted interventions. Social skills training has been shown to have larger effect sizes in a selective and indicated setting (Beelmann & Lösel, 2021). Lastly, indicated prevention should focus on high-risk individuals, like those with a sexual interest in children who might show early signs of pedophilic disorder, e.g., having interpersonal difficulties and distress, are increasingly fantasizing about contacting children, or are tempted to search for CSAM. They should be supported through a therapeutic approach. If an individual has transitioned from preference to disorder, specialized treatment should be offered to manage the disorder in a way that prevents re-offenses, and reduces distress.

For the most part, data on erotic preference for prepubescent and early pubescent children are derived from patients seen in Psychiatry or Sexual Medicine, convicted offenders, or case studies; it is thus skewed toward indicated prevention, which targets a subpopulation that is already showing signs of PHD (Bártová et al., 2021). Indicated preventive treatment efforts address CSA and CSAM separately and involve evidence-based interventions in both populations.

This is closely related to the concepts of primary and secondary prevention when considering the behavioral disorder independently of PH (Knack et al., 2019). Primary prevention involves the complete avoidance of CSA or the use of CSAM, while secondary prevention focuses on preventing the recurrence of CSA or the use of CSAM.

Cognitive Behavioral Therapy, with a relapse prevention approach, which aims to identify high-risk situations and develop alternative coping strategies, is commonly used for CSA offenders. Additionally, rehabilitation-focused approaches seek to equip offenders with the skills to achieve life goals and fulfill basic needs in non-criminal ways (for review, see Sousa et al., 2023).

Meta-analyses highlight that cognitive-behavioral approaches that match the treatment intensity to the offender’s risk of re-offending, target dynamic risk factors (changeable aspects of an individual’s psychology and environment that contribute to criminal behavior), and tailor the treatment to the individual’s learning style, motivation, and abilities are most effective (for an overview, see Deming & Jennings, 2020). A combination of pharmacological and psychological methods addresses PH arousal and dissexuality more comprehensively (McPhail & Olver, 2020). However, the effectiveness of all these approaches is debated (Deming & Jennings, 2020; Sousa et al., 2023).

### Pedophilic and Hebephilic Disorder in the *Dunkelfeld*

Efforts to prevent CSA and the use of CSAM have primarily focused on convicted offenders. However, many offenses remain unknown to judicial institutions and continue to exist in the *Dunkelfeld* (literally “dark field,” referring to offenses that are not officially recorded). Offenders in this group and individuals with PHD who have not acted on their urges are excluded from preventive efforts. The need for broad preventive approaches, including selective and indicated prevention, is increasingly recognized (Beier, 2021). To date, only a few studies have investigated these populations.

One such initiative is the *Prevention Project Dunkelfeld*, launched in 2005 at the Institute of Sexology and Sexual Medicine, Charité - Universitätsmedizin Berlin. The project aims to assist individuals with pedophilia as defined by ICD-10 or pedophilic disorder according to ICD-11 by providing an anonymous therapeutic service for self-motivated individuals, not under criminal investigation. Both offenders unknown to law enforcement who seek to avoid reoffending and individuals with pedophilic sexual preferences who have not offended are eligible. Participants undergo assessments to evaluate their sexual preferences, psychological distress, comorbidities, and risk of offending. The therapeutic goal is to enhance behavioral control and mental health using a modular program that includes behavioral therapy, sexual medicine interventions, pharmacological options, and family involvement, rather than attempting to alter sexual preferences (Beier, 2021). In 2011, the prevention network “Kein Täter werden” (“Don’t offend”) was established, expanding to other German states. Initially funded by the Volkswagen Foundation, the project now receives support from federal and state ministries and is guaranteed financing by health insurance funds through 2025 under the German Social Code, with an annual budget of €5 million. This aligns with EU Directive 2011/92, which mandates prevention programs for individuals at risk of committing sexual offenses against children.

Studies investigating the effects of the Prevention Project Dunkelfeld reported small to moderate changes in dynamic risk factors for CSA over treatment; they recorded relapse rates of 14–20% for CSA and 39–90.6% for CSAM with follow-up periods between 12 and 28 months (Beier et al., 2015; Engel et al., 2019; von Franqué et al., 2023). Evidence for a treatment-specific effect was missing, as was long-term follow-up data (Mokros & Banse, 2019). A comprehensive external evaluation of the network is expected by the end of 2025​​,​​​​ including a randomized controlled trial. Reflecting a scientific bias, data on the general health needs of this population, including mental health, are scarce. Due to limited research, an understanding of the needs of individuals with PHD remains unclear, and strategies for at-risk individuals are inadequate (Levine & Dandamudi, 2016).

### The Present Study

The present study had three objectives: (1) to assess the long-term development of dynamic risk factors; (2) to analyze self-reported CSA/CSAM persistence and recidivism; and (3) to explore the quality of life in a population of self-reporting, help-seeking individuals with PHD.

## Methods

### Participants

**Fig. 1:**
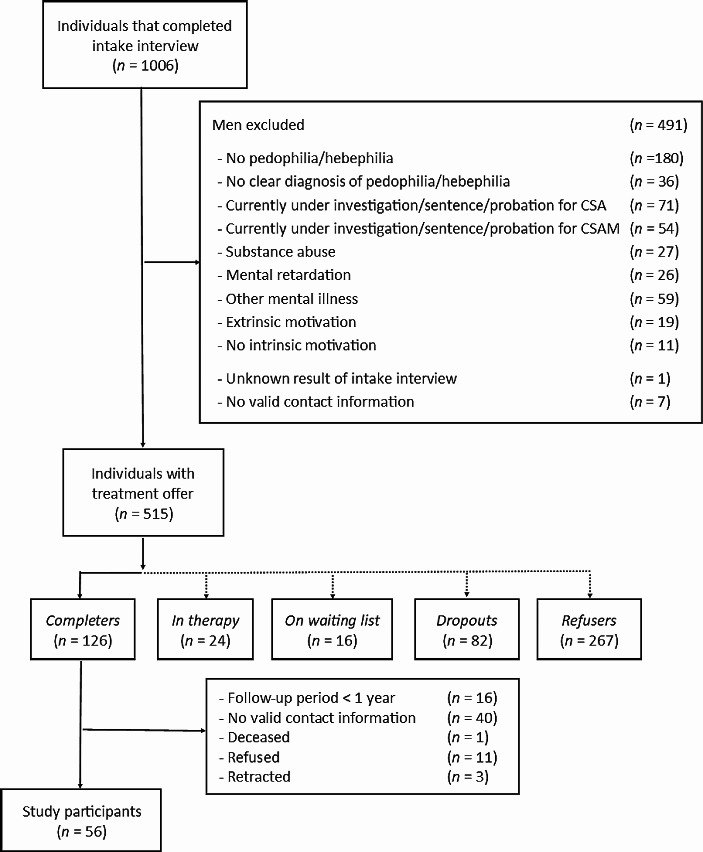
Composition of the sample included. *Note* CSA = child sexual abuse, CSAM = child sexual abuse material

From April 2005 to December 2016, 1,006 individuals were assessed using semi-structured interviews as part of a specialized treatment program for individuals with PHD in *Dunkelfeld*. Interviewees contacted the program via email or phone. Applicants were pseudonymized for the entire process using a numeric code. Treatment eligibility required meeting the DSM-IV-TR (APA, 2000) or DSM-5 (APA, 2013) criteria for pedophilic or hebephilic sexual preference disorder and was verified for reliability by two independent researchers. Individuals under the age of 18 years, those with a current detection status for CSA or CSAM offenses, those with substance abuse, mental retardation, or other mental illnesses impacting treatment responsivity, and those who were externally motivated or lacked intrinsic motivation were excluded from the study. The exclusion process is depicted in Fig. 1. Individuals were invited to participate in the therapy program after a comprehensive evaluation of the inclusion and exclusion criteria. Those who did not meet the criteria were directed to other resources. Participants’ sociodemographic and sexological characteristics and offense history are shown in Table 1. All participants were male.

**Table 1:** Sociodemographic, sexological, and offense history

	*n* (%)
*Age group*	
20–29	2 (3.6)
30–39	14 (25.0)
40–49	20 (35.7)
50–59	16 (28.6)
60–69	4 (7.1)
*Years of education*	
Up to 10 years	20 (35.7)
11 years or more	34 (60.7)
*Employment status*	
Employed	35 (62.5)
Parental leave	1 (1.8)
Unemployed	19 (33.9)
*Social status*	
Single	32 (57.1)
In a relationship	23 (41.1)
Duration of the relationship (*M*, *SD*)	10.15 (9.47)
*Children living in the same household*	
Yes	10 (17.9)
No	46 (82.1)
*Living alone*	
Yes	32 (57.1)
No	23 (41.1)
*Sexual body age preference*	
Pedophilia	16 (28.6)
Hebephilia	15 (26.7)
Both	25 (44.64)
*Sexual orientation*	
Androphilia (attraction toward males)	19 (33.9)
Gynephilia (attraction toward females)	28 (50.5)
Bisexuality (attraction toward both)	9 (16.1)
*Exclusive pedohebephilia*	
Yes	29 (51.8)
No	27 (48.2)
*Paraphilic preferences*	
Specific body parts or objects	8 (14.3)
Transvestic fetishism	8 (14.3)
Exhibitionism	2 (3.6)
Autopedophilia	4 (7.1)
Sadism	7 (12.5)
Masochism	1 (1.8)
Sadomasochism	3 (5.4)
Zoophilia	1 (1.8)
Urophilia	5 (8.9)
Coprophilia	1 (1.8)
Acrotomophilia	1 (1.8)
Macrophilia	1 (1.8)
Urethral sounding	1 (1.8)
*Prior lifetime sexual offenses*	
None	9 (16.1)
CSA only	2 (3.6)
CSAM only	21 (37.5)
Mixed offenses	24 (42.9)
*Previously known to justice*	
Yes	15 (26.79)
For CSA offenses	7 (46.67)
For CSAM offenses	8 (53.33)

*Note* CSA = child sexual abuse, CSAM = child sexual abuse material

### Procedure and Data Collection

The study was approved by the Institutional Ethics Board (vote number: 1754/Si. 251) and ran from June 2015 to August 2017. Out of *n* = 110 eligible participants who completed treatment from 2005 to 2017 and a minimum follow-up period of one year, *n* = 56 consented to follow-up contact (*M*_age_ = 45.5, *SD* = 10.63, age range 24–67). The sample reduction for this study is presented in Fig. 1. All participants gave written informed consent and received a €100 expense allowance.

Treatment lasted 14.38 months on average (*SD* = 5.29, range 10–36). Most participants attended weekly group therapy (94.6%, *n* = 53). Three opted for individual sessions. Treatment followed a cognitive behavioral approach focused on behavioral control, acceptance of sexual preference, and future resilience (Beier, 2021). Following international guidelines (Thibaut et al., 2020), pharmacological treatment was tailored individually according to distress and risk levels (see Amelung et al., 2012). Under German law, participants can safely disclose past offenses without legal consequences. Nearly half of the sample (44.6%) participated in post-treatment support groups. Additional therapies included individual sessions for 10.7% (*n* = 6), couples’ therapy for 42.9% of those in relationships (*n* = 4), and medications to manage sexual urges (SSRIs, androgen antagonists, GnRH agonists) for 28.6% (*n* = 16). At follow-up, six participants were still receiving medication. The average duration of follow-up was 74 months (*SD* = 33.35, range 12–130). The follow-up study involved participant interviews and self-report questionnaires.

Trained psychologists and physicians collected data in a private office using a semi-standardized interview guide. Interviews spanned the complete follow-up period. To reduce bias, the interviewers were not the former therapists. Questionnaires assessed participants’ sexual behaviors over the preceding six months. All but one of the interviews were audio-recorded, averaging 122 min (*SD* = 40.73, range 48–216). The recordings were transcribed verbatim according to the principles of clean verbatim transcription, with dialects and fillers omitted. Data from the pre- and post-treatment questionnaires were used for plausibility checks.

### Measures

As the treatment program follows a cognitive behavioral approach, we restricted our longitudinal analyses to markers of CSA and CSAM behaviors and cognitions surrounding such behaviors. Data on quality of life were assessed only at follow-up. All scales showed excellent internal consistency in this study (see Table 2).

#### CSA and CSAM Use

Interview transcripts were coded to identify the occurrence of CSA and the use of CSAM. Information was compared with data from pre-treatment interviews, treatment notes, and self-report questionnaires. CSA was coded to include all sexual acts performed on or in front of a child under 14 years of age or the instigation of sexual acts by a child on themselves. A distinction was made between offenses that involved physical contact and those that did not (e.g., indecent exposure). CSAM use was considered separately. The severity of the materials was assessed at each measurement point using the most severe category. Images were categorized on a reduced version of the COPINE scale (“Combating Paedophile Information Networks in Europe”), a rating system used in the UK to categorize the severity of child abuse images (Quayle, 2008). The original 10 COPINE levels were condensed into six categories, each representing an increasing degree of severity: Level 1, indicative non-sexualized images of children in everyday situations; Level 2, non-erotic depictions of nude children; Level 3, depictions of children in erotic poses; Level 4, which includes explicit posing of children with an emphasis on genitalia, sexual activity between children, or solo masturbation by a child; Level 5, depicting sexual activity with an adult, and Level 6, depicting sadistic acts or bestiality. Instances of accessing materials categorized as levels 3–6 were deemed CSAM (re)offenses. A researcher not involved in the treatment program performed the coding. As a reference for inter-rater reliability, 32 interviews were additionally coded by the interviewers with an average rater agreement of 72.1%. In instances where the assessment diverged, priority was given to the interviewers’ assessment. Missing data due to inaudible recordings were supplemented with data from self-report questionnaires.

#### Cognitive Victim Empathy

The Cognitive Empathy Deficits for Children Scale (CEDCS; modified German version) is a 30-item instrument used to measure cognitive empathy toward child victims using two scenarios: a child is sexually abused by an unknown person; a (real or fantasized) child is sexually abused by the respondent. Respondents indicated their perception of how likely the child was to experience different negative thoughts, feelings, and behavioral problems during and after sexual contact using a 5-point Likert scale ranging from 1 (not at all) to 5 (very). A sample item is “The child has feelings of guilt.” Higher values reflect a more significant empathy deficit. No community norms are given for this scale.

#### CSA-Supportive Attitudes

The Bumby Molest Scale (BMS; modified German version) is a 38-item instrument used to assess CSA-supportive views on a 4-point Likert scale ranging from 1 (strongly disagree) to 4 (strongly agree). A sample item is “Sexual activity with children can help the child learn about sex.” Higher scores indicate stronger CSA-supportive attitudes. The comparative mean of 30 men from the community is given as *M* = 51.8, *SD* = 10.4 (Marshall et al., 2003).

#### CSAM-Supportive Attitudes

The Attitudes Related to Sexually Explicit and Nonexplicit Images of Children Questionnaire (ASENIC; Neutze, unpublished) includes 28 items scored on a 4-point Likert scale ranging from 1 (strongly disagree) to 4 (strongly agree). A sample item is, “Children and adolescents take part in pornographic movies and images out of curiosity.” Higher scores indicate stronger CSAM-supportive attitudes. No community norms are given for this scale.

#### Quality of Life

The EUROPE Health Interview Survey–Quality of Life (EUROHIS-QOL) is an 8-item index. Items are rated on a 5-point Likert scale ranging from 1 (not at all) to 5 (completely). A sample item is, “How satisfied are you with the conditions of your living place?” Higher scores indicate a better quality of life. Male norm values in Germany (*n* = 1166) are *M* = 31.3, *SD* = 3.8.

### Data Analysis

Analyses were conducted using the IBM SPSS Statistics Software. The type I error level was set at *alpha* < 0.05. Fisher’s Exact Test and t-tests were used to test for group differences. Dependent t-tests and repeated measures analysis of variance were used to assess changes over time. Missing values occurred because of to changes in assessment throughout the project. We accepted 10% missing values for hot-deck multiple imputation (2.2% of data). Cases exceeding 10% of missing values per scale were omitted from the analyses (2.0% of data). Comparisons of questionnaire data with comparative means or norms were conducted using independent t-tests with the online calculator at https://www.graphpad.com/quickcalcs/ttest1.cfm. Estimates of confidence intervals for recidivism rates were computed using the Wilson interval implemented in the online calculator at https://epitools.ausvet.com.au/ciproportion.

## Results

An initial exploratory examination of the reasons for seeking help revealed that nine men entered the treatment program shortly after committing a CSA offense and four because they had previously been discovered by authorities or caught by their partner in a CSAM offense. When asked what they hoped to gain from treatment, the most common responses were that they wanted to gain control over their sexual preference, not re-offend, be cured of pedophilia, gain clarity about their sexual preference, or learn to accept their sexual preference. Some said they came because they could not cope with their sexual preference or because of external social pressure. One participant said:

I wanted some kind of healing. That somehow everything disappears. All the thoughts, all the fantasies and everything, and that I could start all over again.

### CSAM Use

The content and frequencies of the overall CSAM consumption are presented in Table 2 for each measurement point. Post-treatment, the number of participants who used CSAM dropped to *n* = 35 (62.5%, 95%-CI: 49.4–74.0%) from *n* = 46 at the pre-treatment stage. Rates of use increased at follow-up (*n* = 42, 75%, 95%-CI: 62.3–84.5%), but with a shift from severe categories like “sadism or bestiality” and “assault” to less severe categories like “erotic posing” and “explicit posing or sexual activity” compared with pre-treatment levels. One of the 10 participants who had not used CSAM prior to treatment started consuming material occasionally during follow-up. Recidivism rates reached 76.1% (95%-CI: 62.1–86.1%) at post-treatment and 89.1% (95%-CI: 77.0–95.3%) at follow-up.

**Table 2:** Content and frequency of overall CSAM consumption

	Pre-treatment	Post-treatment	Follow-up
	*n* (%)	*n* (%)	*n* (%)
*Highest level of severity of material used*			
0—No material at all	4 (7.1)	11 (19.6)	8 (14.3)
1—Indicative	1 (1.8)	3 (5.4)	1 (1.8)
2—Nude	5 (8.9)	7 (12.5)	5 (8.9)
3—Erotic posing	1 (1.8)	3 (5.4)	11 (19.6)
4—Explicit posing or sexual activity	7 (12.5)	9 (16.1)	11 (19.6)
5—Assault	25 (44.6)	12 (21.4)	14 (25.0)
6—Sadism or bestiality	9 (16.1)	5 (8.9)	4 (7.1)
CSAM consumption, but severity content unknown	4 (7.1)	6 (10.7)	2 (3.6)
Number of CSAM consumers (with erotic posing)	46 (82.1)	35 (62.5)	42 (75.0)
*Highest frequency of CSAM consumption*			
Few occasions	9 (16.1)	8 (14.3)	20 (35.7)
Monthly	5 (8.9)	1 (1.8)	4 (7.1)
Weekly	5 (8.9)	0 (0)	4 (7.1)
Daily	9 (16.1)	1 (1.8)	1 (1.8)
Unknown	18 (32.1)	25 (44.6)	13 (23.2)

*Note* CSAM = child sexual abuse material. Severity levels 3–6 are considered to be CSAM. Percentages refer to the entire population under observation (*N* = 56)

Of those who persisted in using CSAM, 31.0% (*n* = 13) resorted to similar material, whereas 45.2% reported a reduction in the severity of the content consumed (*n* = 19) and 5.0% (*n* = 2) an increase in the severity of the content consumed. Data from eight participants were missing. This is an example of a response from a former participant in the program who used CSAM again during the follow-up period:

If I imagine a real-life encounter where nothing happened, if I saw a boy on the bus or something and then imagine him naked at home, then nothing happened. I still think it’s very dirty. I feel like I’m abusing him on a fantasy level. I find that inappropriate. The images are more abstract for me, even though something really happened. And I find that psychologically logical but abstract. And somehow absurd, tragically absurd.

### CSA Behavior

Participants without prior offenses reported no CSA offenses during the observation period. Approximately half of the sample reported committing CSA before treatment (*n* = 26, 46.4%). Two (7.7%, 95%-CI: 2.1–24.1%) recidivated during follow-up. The first case was a male individual with an exclusive PHD oriented toward girls. He reported having sexually abused his daughter on 47 occasions beginning at the age of four. The abuse started before entering treatment and occurred once during treatment. During follow-up, while on testosterone-lowering medication (cyproterone acetate), he sexually abused his girlfriend’s eight-year-old son; at the time, he reported intimacy deprivation, depression, and social withdrawal. The second case was a male participant diagnosed with exclusive pedophilic disorder oriented toward boys. He reported a history of offenses prior to treatment, including performing fellatio on a 10-year-old boy. He had a low response to therapy. At follow-up, it became known that he had concealed information crucial to the treatment. He had just joined the aftercare group, hiding the fact that he was currently under probation for CSA. During the follow-up interview, he reported masturbating and engaging in passive anal intercourse with a boy, allegedly 15 years of age, that he had picked up on the street in return for payment. Piecemeal revelations during the follow-up interview gave the impression that the boy was probably under the age of 14. An unknown party independent of the project filed charges for the said behavior. Thus, he was excluded from the program and received further treatment in an outpatient clinic.

### Questionnaire Data

Compared to mean values from a community sample (*M* = 68.3, *SD* = 15.8), participants showed increased CSA supportive attitudes at all three points of measurement, as revealed by two-tailed *t*-tests at pre-treatment (*t*(51) = 4.62, *p* < .001, Cohen’s *d* = 1.26, 95% CI: 0.68–1.85), post-treatment (*t*(79) = 3.88, *p* < .001, Cohen’s *d* = 0.89 [0.42–1.36]), and follow-up (*t*(65) = 4.40, *p* < .001, Cohen’s *d* = 1.08 [0.56–1.59]). Cognitive victim empathy deficits and CSA supportive attitudes showed a significant decrease during treatment with a small effect size. In contrast, supportive attitudes toward CSAM significantly decreased with a large effect size. Supportive attitudes toward CSAM remained significantly lower at follow-up than at pre-treatment. Details are shown in Table 3. Quality of life at follow-up was significantly lower than community norms (*M* = 31.3, *SD* = 3.8) with a medium effect size (*t*(1212) = 4.08, *p* < .001, Cohen’s *d* = 0.60 [0.31–0.89]). One participant who was using CSAM daily before therapy and had a number of relapses in the four years of follow-up after stopping an anti-androgen medication reported:

I am finally alive. Before, I felt like I could never really live, never really be free, because I never accepted myself for who I am. In my eyes, I was always the monster back then because everyone said that people like that are monsters, and I kept writing that in my own book that I am the monster. And it was only through group therapy and therapy that I learned to accept who I am. And I think it was really at that moment that I said to myself for the first time: “Okay, that’s the way it is, but you’re not the monster that everyone here always says somehow, that you always hear everywhere. You’re not the monster. You’re not the perpetrator. It’s just the way it is. You didn’t choose it; life isn’t a pony farm.” And that’s how it is, and since then, I’ve been able to clear my head and really live. So a lot, a lot, a lot has changed since the therapy and since the injections, which I could never have imagined back then, that I could really be - well, I’ll say it now - happy. I would never have thought that. Things have happened since then that I would never have dreamed of.

**Table 3:** Psychometric measures

	Pre-treatment			Post-treatment				Follow-up			
	α	*n*	*M (SD)*	α	*n*	*M (SD)*	vs. pre-treatment	α	*n*	*M (SD)*	vs. pre-treatment
Cognitive victim empathy deficits	0.98	53	76.6 (32.4)	0.98	51	66.2 (27.9)	*t*(49) = 2.38* *d* = 0.34 [0.05–0.62]	0.96	26	67.0 (22.8)	*t*(24) = 1.40 *d* = 0.28 [− 0.12–0.68]
CSA-supportive attitudes	0.99	24	68.3 (15.8)	0.95	51	64.7 (16.4)	*t*(21) = 2.16* *d* = 0.46 [0.15–0.90]	0.94	37	67.0 (16.5)	*t*(18) = 0.45 *d* = 0.10 [− 0.35–0.55]
CSAM-supportive attitudes	0.94	37	50.1 (13.7)	0.94	36	42.9 (12.2)	*t*(31) = 6.17*** *d* = 1.09 [0.65–1.53]	0.92	28	43.7 (12.0)	*t*(20) = 4.49*** *d* = 0.98 [0.45; 1.50]
Quality of Life								0.80	48	29.0 (4.94)	

*Note* See the method section for instruments used for the assessment of psychological variables; CSA = child sexual abuse; CSAM = child sexual abuse material. Note that missing values were unevenly distributed between assessment points, leading to differentially reduced samples for dependent t-tests. The effect size is given as Cohen’s *d* ± 95%-CI.

* *p* < .05; ** *p* < .01; *** *p* < .001

## Discussion

This study investigated the long-term development of dynamic risk factors for child sexual abuse, examining changes in behaviors and cognitions about CSA and the use of CSAM in self-referring, help-seeking individuals with PHD in the *Dunkelfeld*, i.e., outside a judicial context. In this study, 46.4% of the participants had engaged in CSA before treatment, of whom two recidivated. No new CSA offenses occurred among participants who had no prior history of CSA offending. For acts related to CSAM, a reduction in use was recorded throughout treatment, but the majority recidivated until follow-up; one individual reported first-time use. Content severity was slightly reduced throughout the study period. Participants showed increased CSA-supportive attitudes at all assessment points compared to community norms and impaired quality of life at follow-up. CSA supportive attitudes and cognitive victim empathy deficits decreased throughout therapy; however, they returned to previous levels at follow-up. CSAM-supportive attitudes did not rebound from post-treatment until follow-up.

Regarding conduct related to CSA, the 95%CI showed the self-reported recidivism rate to be comparable to rates of officially known recidivism established meta-analytically in convicted sexual offenders (Hanson & Morton-Bourgon, 2004; Schmucker & Lösel, 2017). Comparison data of self-reported recidivism involving undetected offenses is scarce. Beier (1998), in his study of convicted offenders, found rates between 50% for non-exclusive pedophilia and 80% for exclusive pedophilia, including unreported *Dunkelfeld* offenses. A recent analysis found a self-reported recidivism rate of 14% (von Franqué et al., 2023). The 7.7% recorded in this study thus sits at the lower end of this distribution. Notably, the ratio of self-reported sexual offenses to officially known offenses is unclear. While confidentiality laws in Germany and its explicit mention to participants in this study may have increased the willingness to admit past sexual offenses, there still is ample ground for individuals to conceal past sexual offenses in a one-on-one interview setting with an unknown interviewer, leaving room for speculation about potentially unrevealed additional offenses committed by the study sample. The numbers presented here may thus underestimate the number of offenses committed.

Similar to Beier et al. (2015), this study found that most individuals with a history of using CSAM recidivate. There is a striking difference between these numbers and rates of recidivism in convicted offenders, e.g., the 2–13% to thirteen per cent recidivism on average reported by Helmus and colleagues (Helmus et al., 2024). These differences may be attributed to greater openness in a non-judicial context. Moreover, direct encounters with law enforcement, such as investigations, arrests, or other forms of legal intervention, may deter individuals from continuing to access or distribute CSAM (Steel et al., 2022).

Compared with other results from *Dunkelfeld*, *w*hich reported a recidivism rate of 39% (95%-CI 30.8–47.2%, own calculation), our recidivism rate is still high (von Franqué et al., 2023). Since recidivism rates were recorded over roughly four years, and assuming that the sexual preference for prepubescent and/or early pubescent children remains largely stable over time (Grundmann et al., 2016), it is possible to infer that the risk of recidivating increases with time.

This interpretation becomes plausible when accounting for changes in offense-supportive cognitions over time. While a reduction in said cognitions was apparent over the treatment phase, this effect faded toward follow-up, potentially facilitating re-engagement in abusive behaviors. Parallels with settings showing initial symptom improvement followed by deterioration are pertinent (Cuijpers, 2019). Given that entrenched dysfunctional interaction patterns are highly prevalent in the studied population (Gerwinn et al., 2018), this type of development, i.e., an improvement of dysfunctional cognitions through therapy and its deterioration over time after therapy, is not surprising. In addition, the assumable stability of PH sexual impulses and fantasies might have driven this deterioration: The new, therapeutically acquired cognitions will cause a cognitive dissonance with the experienced or expected sexual pleasure. The deterioration of offense-supportive cognitions and cognitive victim empathy may have helped participants reduce this dissonance, rendering recidivism a more likely scenario.

Criticism of the *Dunkelfeld* approach has voiced concerns about possible iatrogenic (treatment-induced) effects that may increase the likelihood of re-offending rather than reducing it (König, 2023). One major objection refers to findings where the iatrogenic effect was seen in low-risk sexual offenders. Accordingly, a recent analysis found *Dunkelfeld* individuals with pedophilic disorder to exhibit low to moderate actuarial risk levels (von Franqué et al., 2023). Simultaneously, the study found recidivism rates concerning CSA typical of the average re-offending rates of convicted offenders and even increased recidivism rates concerning CSAM offenses. Remarkably, those re-offenses were unrelated to the actuarial risk assessment scores. Actuarial risk assessment tools thus appear to not apply in *Dunkelfeld*, and a discussion of iatrogenic effects in actuarially appraised “low-risk offenders” will need to wait until risk in *Dunkelfeld* is better understood. Furthermore, naturalistic observational studies have shown high rates of sexual recidivism in convicted pedophilic sexual offenders against children, most of whom are in *Dunkelfeld* (see e.g., Beier, 1998). The assertion that 0% reconvictions in individuals self-reporting sexual offenses against children represents the lowest possible forensic risk may be logically true but seems cynical from a victim perspective. Iatrogenic effects cannot thus be completely ruled out due to the lack of a control group, whereas an ongoing risk due to the persistent erotic attraction for prepubescent and/or early pubescent children appears as an equally plausible explanation for the observed recidivism.

### Limitations

The purely observational nature of this study and the lack of a control group impede causal conclusions; therefore, well-controlled studies are imperative. Regarding data collection, variations in interview length may have contributed to the missing values. Future studies should implement standardized interviews that follow specific coding rules to minimize data gaps. Given the treatment program’s commitment to anonymity, the data relies solely on self-reported information without access to official criminal records. Although therapists assured participants of confidentiality, participants may have under-reported problematic behavior and, therefore, diminished the impact of addressing ongoing behavioral problems. Thus, it is skewed toward indicated prevention, which targets a subpopulation already showing signs of PHD. Finally, the anonymous nature of this project hindered our ability to obtain the contact information of all eligible candidates. Despite our inability to contact 36% of eligible participants because of missing contact information of all eligible candidates, those contacted successfully exhibited an 80% response rate, exceeding most follow-up studies.

## Conclusion

In a long-term follow-up analysis of a sample of self-referring, help-seeking individuals with PHD undergoing treatment in *Dunkelfeld*, we found recidivism rates of 7.7% with CSA and 89.1% with CSAM. At the same time, 50 individuals remained abstinent of CSA but only 5 of CSAM. Changes over treatment in the desired direction in cognitive empathy, CSA and CSAM supportive attitudes, but only the latter effect remained until follow-up. Quality of life was reduced compared with community norms.

Our data suggest that PHD in the *Dunkelfeld* is associated with a persistent risk for sexually abusive behavior, which differs from data collected from convicted offenders using criminal records, especially concerning CSAM use. Therapy for individuals with PHD in the *Dunkelfeld* may alter cognitions relevant to the risk of engaging in sexually abusive behaviors that remit over time without therapy. In addition, PHD in *Dunkelfeld* patients may be associated with impairments in general quality of life. Assuming sexual interests for prepubescent and/or early pubescent children are stable, long-term risks of developing internalizing (withdrawal, non-disclosure) or externalizing (behavioral problems, CSA, CSAM use) symptoms may exist in this population. As in other chronic conditions, early detection, targeted treatments and a well-founded aftercare concept should be offered for PHD, including specialized psychotherapy and effective pharmacological options to reduce sexual needs, e.g. androgen deprivation treatment (Landgren et al., 2020; Beier, 2021).

In conclusion, while preventive treatment for self-motivated individuals with PHD shows promise in reducing dynamic risk factors, the effects may not be sustained long-term without ongoing care. More research is needed to optimize therapeutic interventions, identify individuals at the highest risk of reoffending, and establish integrated public health strategies to prevent child sexual abuse and exploitation in this population. Collaboration between mental health professionals, public health officials, policymakers, and the justice system will be key to tackling this complex issue and protecting children from sexual violence.
